# The IUPHAR/BPS Guide to PHARMACOLOGY in 2026

**DOI:** 10.1093/nar/gkaf1067

**Published:** 2025-10-29

**Authors:** Simon D Harding, Jane F Armstrong, Elena Faccenda, Christopher Southan, Stephen P H Alexander, Anthony P Davenport, Michael Spedding, Jamie A Davies

**Affiliations:** Institute for Neuroscience and Cardiovascular Research, University of Edinburgh, Edinburgh EH8 9XD, United Kingdom; Institute for Neuroscience and Cardiovascular Research, University of Edinburgh, Edinburgh EH8 9XD, United Kingdom; Institute for Neuroscience and Cardiovascular Research, University of Edinburgh, Edinburgh EH8 9XD, United Kingdom; Institute for Neuroscience and Cardiovascular Research, University of Edinburgh, Edinburgh EH8 9XD, United Kingdom; School of Life Sciences, University of Nottingham Medical School, Nottingham NG7 2UH, United Kingdom; Experimental Medicine and Immunotherapeutics, University of Cambridge, Cambridge CB2 0QQ, United Kingdom; Spedding Research Solutions SAS, Le Vésinet 78110, France; Institute for Neuroscience and Cardiovascular Research, University of Edinburgh, Edinburgh EH8 9XD, United Kingdom

## Abstract

The IUPHAR/BPS Guide to PHARMACOLOGY (GtoPdb, https://www.guidetopharmacology.org) is an open-access database of expert-curated, high-quality pharmacological data. It provides succinct overviews and key references for pharmacological targets and their recommended experimental ligands, alongside detailed information about ligand–target activities. It includes 3103 protein targets and 13 260 ligand molecules, including approved drugs, small molecules, peptides, and antibodies. Here, we report recent improvements to the resource and describe expansion in content over the seven database releases made during the last two years. We describe developments in antibacterial pharmacology, work that has been supported through our collaboration with AntibioticDB and Global Antibiotic Research and Development Partnership. We discuss recent areas of curation, including natural products and nucleic acids, and describe an improved presentation of our approved drugs set that contains new therapeutic targets and ligands that are proposed as modulators with clinical potential. This is a useful new resource, both in being continuously updated and having coverage across multiple approval agencies. Maintaining strong links to external resources, such as PubChem, remains an important feature of GtoPdb, and here we highlight their value and utility in being a FAIR-compliant resource. We also present a comparison of our data coverage with ChEMBL and BindingDB.

## Graphical Abstract

**Graphical Abstract:**
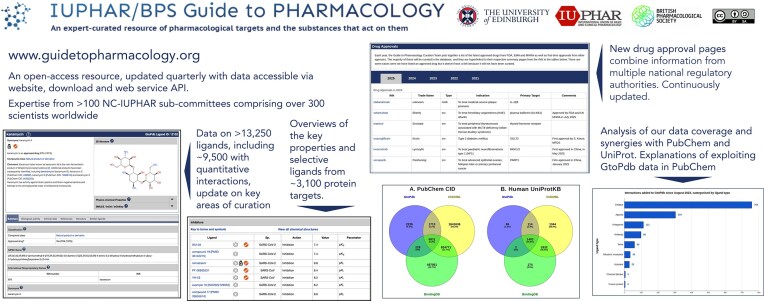

## Introduction

The Guide to PHARMACOLOGY (GtoPdb), established in 2011, is developed by the International Union of Basic and Clinical Pharmacology (IUPHAR) and the British Pharmacological Society (BPS). It originated from IUPHAR-DB, a former resource focused on receptors and channels that was first compiled in 2003 [1–3] and from the BPS “Guide to Receptors and Channels” [4], a compendium of a wider range of targets than those originally covered in IUPHAR-DB. GtoPdb has continued to expand its coverage of target families and quantitative target–ligand interactions, with guidance and oversight maintained through the Nomenclature and Standards Committee of the International Union of Basic and Clinical Pharmacology (NC-IUPHAR). This has included Wellcome Trust-funded expansions into the data-supported druggable human proteome [5] and immunopharmacology, with the IUPHAR Guide to IMMUNOPHARMACOLOGY (GtoImmuPdb, https://www.guidetoimmunopharmacology.org) [6–8]. There has also been expansion into malaria pharmacology with the Guide to MALARIA PHARMACOLOGY (GtoMPdb, https://www.guidetomalariapharmacology.org) [9], a project funded as a collaboration between IUPHAR and Medicines for Malaria Venture (MMV,https://www.mmv.org/). Overall, throughout the course of its development, GtoPdb has included input from cumulatively over 1000 scientists from 109 NC-IUPHAR subcommittees [10] and presently the database has around 325 active contributors. Analysis of structure/activity on drug targets by machine learning requires *in vitro* bioactivity modulation parameters across multiple (on or off) targets. Variables in such measurements make absolute comparative values difficult to determine but we select by expert curation from quality publications [1–12].

GtoPdb is also the source of the Concise GtoPdb, a biennial publication that is a tabular extract of the database providing concise overviews of the key properties of nearly 1900 human drug targets with an emphasis on selective pharmacology [11]. We currently collaborate with AntibioticDB (ADB, https://www.antibioticdb.com) [13], in a project supported by the Global Antibiotic Research and Development Partnership (GARDP, https://gardp.org/) [14, 15]. Antimicrobial resistance is one of the top threats to global health (https://www.who.int/news-room/fact-sheets/detail/antimicrobial-resistance), and it has been estimated that nearly 1.3 million people die annually as a result of drug-resistant bacteria [16–18]. ADB catalogs and describes known antibacterial agents, to serve as a leading resource for future research and development of antibacterial therapeutics. Our collaboration with ADB continues to strengthen both resources, extending antibacterial compound coverage in GtoPdb, building reciprocal links between the two resources, and providing key pharmacological data for ADB. Since our last update in 2024 [19], we have made seven database releases. In this paper, we describe the key recent curatorial updates for ligands, targets, and interactions in the database, in particular in relation to our collaboration with ADB. We also describe how GtoPdb is FAIR-compliant, with our data being easily accessible to users and how GtoPdb links with external resources, such as PubChem [20]. We discuss how this close integration can be exploited by users, bringing added value to the data.

## Guide to PHARMACOLOGY database updates

### Summary of content and curation

Curation and database development is led by the GtoPdb Curation Team (https://www.guidetopharmacology.org/about.jsp#curation) based at the University of Edinburgh, UK. Our specialized and stringent approach to curation involves human expertise at all stages and is conducted in collaboration with NC-IUPHAR subcommittees. GtoPdb’s strength lies in this discerning, independent and expert-led curation and the prioritization of data validated from independent sources. A valuable feature of the GtoPdb is the speed with which new content is released to the live website. We provide four releases each year, which offer our users access to information about new therapeutic strategies in a timely manner. Table 1 shows a summary of targets, ligands, and interactions curated in the latest release of GtoPdb (v2025.2, 18 June 25). Online database reports, compiled to accompany our biannual IUPHAR/BPS combined meetings, provide additional details (https://www.guidetopharmacology.org/download.jsp#db_reports).

**Table 1.** Guide to PHARMACOLOGY data counts for targets, ligands, and interactions from database release 2025.2 (June 2025)

**A. Target class content: Human UniProtKB accession counts**	
GPCRs	399 (0)
Nuclear hormone receptors	48 (0)
Catalytic receptors	253 (0)
Ion channels	278 (0)
Transporters	555 (0)
Enzymes	1315 (+38)
Other proteins	278 (+26)
Total number of targets	3103 (+64)
	
**B. Ligand category counts**	
Synthetic organics	9329 (+778)
Metabolites	513 (+6)
Endogenous peptides	824 (+11)
Other peptides including synthetic peptides	1545 (+51)
Natural products	521 (+118)
Antibodies	447 (+93)
Inorganics	39 (0)
Approved drugs	2120 (+202)
Withdrawn drugs	116 (+7)
Coronavirus	103 (+3)
Antibacterials	668 (+197)
WHO essential list	305 (+4)
Antimalarial	143 (+7)
SARS-CoV-2 antiviral agents^b^	194^a^
Ligands with INNs	3734 (+423)
PubChem CIDs	10 772
PubChem SIDs	13 260
Total number of ligands	13 260 (+1124)
	
**C. Interaction counts**	
Human targets with ligand interactions	2027 (+80)
Human targets with quantitative ligand interactions	1767 (+72)
Human targets with approved drug interactions	757 (+25)
Primary targets^c^ with approved drug interactions	355 (+8)
Ligands with target interactions	10 835 (+698)
Ligands with quantitative interactionsApproved drugs with quantitative interaction	9571 (+612) 1191 (+84)
Ligands with clinical use summariesApproved drugs with clinical use summaries	4053 (+559)2119 (+209)
Total quantitative interaction	21 643^a^
Unique target–ligand pairs	19 613^a^
Number of binding constants	52 850 (+1505)
References	47 380 (+3383)

^a^No comparative data as the figure was not included in the previous NAR update.

^b^A class that modulate CoV protein targets.

^c^Primary target indicates the dominant MMoA.

The changes from the 2023.2 (August 2023) release are shown in parenthesis. Categories are not mutually exclusive, and targets and ligands can fall into more than one.

### Curation criteria

Our curatorial criteria will only add ligands to GtoPdb when we can confirm name-to-structure associations, find citable evidence that confirms molecular mechanism of action (MMoA) and a source of quantitative interaction data. Including selected sources outside peer-reviewed literature, while maintaining our curatorial stringency, allows the GtoPdb to accumulate novel ligand data at a faster pace than could be achieved by relying on published articles alone. One of our quality hallmarks is stringent selection not only of the papers from which activity relationships are extracted but also references chosen to be cited by expert contributors on NC-IUPHAR subcommittees, in order to give pharmacological contextual support to ligands and targets. This has become increasingly challenging as Europe PubMed Central (EPMC) approaches 46 million records. A recent publication entitled “The entities enabling scientific fraud at scale are large, resilient, and growing rapidly” [21] also brings the problem into sharp perspective. One approach we use to ameliorate this is to regularly intersect our reference list with the “Retraction” tags in PubMed and EPMC, now numbering ∼28 500. Publications in any of our reference lists that are subsequently retracted are identified with warning tags. The evolution of novel drug modalities brings challenges to our curatorial criteria, which, due to a number of factors, mean that ligands may not have curated quantitative interactions with their primary target(s). For example, some of the newest drug classes do not lend themselves to measurements of classical quantitative drug–target pharmacological interactions (nucleic acid type drugs and protein degraders), and for others such data are not readily available (monoclonal antibodies and antimicrobials). In cases such as these, the data do not fit our database schema, with the information captured in comments rather than in our interaction tables. In conjunction with our quarterly GtoPdb release schedule, our curatorial approach facilitates the rapid dissemination of curated, evidence-based pharmacology content to the wider scientific community, both on the GtoPdb website and via our PubChem updates.

### Ligands

Since our last update [19], we have added 1142 new ligands to the database. A summary of these recent additions is shown in Table 2. This highlights the addition of 183 antibacterial compounds, 144 natural products (NPs), and 33 nucleic acid–class ligands, reflecting the main areas of recent curation, particularly through collaborations with ADB and SIF, over the last two years. Across all ligand categories, a total of 128 new approved drugs were added. Overall, 53% (591) of new ligands have quantitative data on their interactions with their targets. Table 2 also indicates where ligands that were already curated in GtoPdb have been updated under each category.

**Table 2.** Summary of new ligands added to GtoPdb in the 2025.2 database release (18th June 2025)

	New ligands	Updated ligands	Total ligands
Approved drugs	128	74	2120
Antibacterials	183	14	668
Ligands with quantitative interaction data	591	3	9591
Nucleic acids^a^	33	9	42
Natural products^b^	144	−26	521
All ligands	1097	–	13260

a Nine previously existing ligands were re-classified as nucleic acids

b Twenty-six previously existing NPs were re-classified as part of our collaboration with SIF

“New ligands” column shows count of new ligands for each category; “Updated ligands” shows count of existing ligands, already curated in GtoPdb, now included in the categories.

New ligands are curated from several sources:

- Ligands submitted as part of database updates from our NC-IUPHAR target family subcommittees.
- Journals with a high density of past entries. Foremost of these is the Journal of Medicinal Chemistry where the inclusion of SMILES for lead compounds is particularly useful for curation. We also monitor social media for relevant new papers.
- New ligands from the proposed INN lists, in conjunction with new target identifications as outlined below. Curators interrogate various sources (PubChem, ChEMBL, patents, and pharmaceutical company pipeline resources) to map the INNs to SMILES, company research codes, interaction data, and where relevant, clinical potential.
- Ligands with antibacterial activity, curated as part of our collaboration with ADB.
- Inspection of the open Drug Hunter (https://drughunter.com) and Dalriada (https://dalriadatx.com/articles/) monthly lists of novel clinical leads plus their “first disclosures” from sessions at scientific meetings. These are only curated if the *in vitro* activity is openly provenanced (even if patent-only) and we update our records with eventual journal publications.
- New ligands for existing targets are extracted from these same resources. Examples include novel chemotypes for inhibitors, ligands with novel or alternate mechanisms of action (exemplified by protein degrader molecules as alternatives to small molecule inhibitors or monoclonal antibodies, and nucleic acid–class drugs that are used to reduce expression of disease-associated proteins), and first drug approvals for specific diseases or conditions [e.g. suzetrigine (https://www.guidetopharmacology.org/GRAC/LigandDisplayForward?ligandId=12630), the first oral, selective Na_V_1.8 (SCN10A) allosteric inhibitor to be approved to reduce pain].
- Information regarding the approval status of drugs directly from the FDA (https://www.fda.gov/drugs/novel-drug-approvals-fda/novel-drug-approvals-2025), EMA (https://www.ema.europa.eu/en/medicines), other accessible national agencies’ online portals, and from the “Drugs” journal (https://link.springer.com/journal/40265).
- New compounds of interest highlighted by our collaboration with the Probes & Drugs database and the Chemical Probes Portal (for which one of us, CS is a reviewer) [22].
- Authors of papers in the BJP family of journals (https://www.bps.ac.uk/publishing). The BJP instructions for authors recommend linking ligands and targets to our GtoPdb entries in the eventual on-line HTML and PDF manuscripts. A small number of these turn out to be novel, for which we curate a new entry as part of the author submission process and update with the assigned BJP PMID when it becomes available.

To extract chemistry from papers and patents, the curators use open tools such as DECIMER (https://decimer.ai/) [23] to generate SMILES from 2D chemical images and OPSIN (EMBL-EBI; https://opsin.ch.cam.ac.uk/) [24] to convert IUPAC names to SMILES. All ligand SMILES notations are entered into GtoPdb as the primary chemical structure identifier and are used to map our ligands to PubChem and ChEMBL [25]. We also include International Chemical Identifiers (InChI) (https://www.inchi-trust.org/about-the-inchi-standard/) [26, 27], both standard InChI and InChIKeys, for expanded interoperability and searching options.

### Drug approval pages

**Figure 1.**
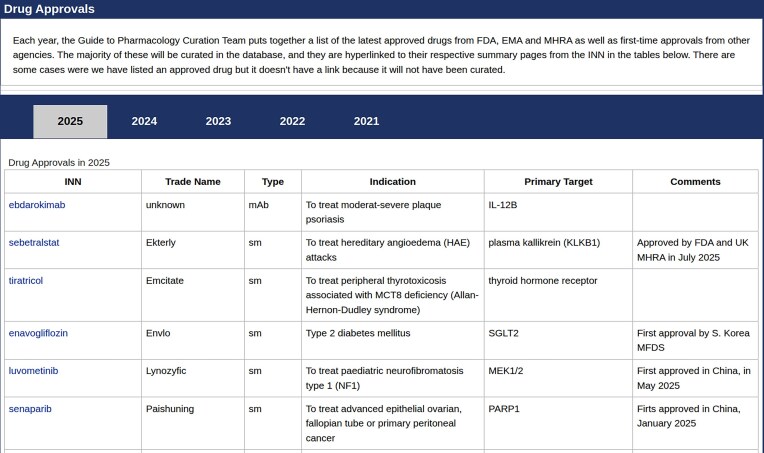
Screenshot of the new drug approval page on the GtoPdb website: https://www.guidetopharmacology.org/GRAC/DrugApprovalsForward.

The GtoPdb curators continually track the latest drug approvals from the FDA, EMA, and MHRA as well as first-time approvals from other agencies. We have now developed a specific page (https://www.guidetopharmacology.org/GRAC/DrugApprovalsForward) on the website that records details of drug approvals, making this information more readily available to users. This new page lists these drug approvals in tables, organised by year of approval (Fig. 1). Drugs that are curated in the database are hyperlinked to their respective ligand summary pages from the INN in the table. Some may appear in these tables that are not curated into the database (and therefore do not have a link to a GtoPdb webpage) because they don’t meet our curation criteria. As we combine information on drug approvals from multiple national regulatory authorities, we are able to provide our users with easy access to lists of approved drugs that are more extensive than publications that focus on approvals by specific national agencies [28–30] (NMPA, EMA, and FDA).

### Targets

As a general rule, GtoPdb curators add new protein targets when there is robust evidence of direct pharmacological modulation at therapeutically relevant concentrations and accompanying *in vivo* data implying potential clinical utility. Targets in GtoPdb use a UniProtKB/SwissProt [31] accession as their primary identifier and are organised into hierarchical target families. Information about new protein targets is extracted from a number of sources:

- Peer reviewed literature, including primary medicinal chemistry journals and clinical and therapeutic journals, are the principal resources for data curation.
- From patents, which includes those suggested to us through our collaboration with BindingDB [32].
- The WHO publishes two lists of proposed INNs each year from which new targets are identified via the mechanism of action provided for each compound.
- Suggestions from speakers who are invited to NC-IUPHAR meetings, that reflect evolving pharmacology and therapeutic potential of ligand–target pairings.

Since our last NAR review and update, we have added 64 new protein targets, the majority of which were added to the Enzymes or Other Proteins sections of the GtoPdb. Several were added as part of our NPs project, as there was published evidence that they are direct molecular targets of naturally occurring compounds. Others were added based on evidence of clinical potential, across a range of therapeutic areas. For example, 22 proteins were added as experimental or investigational oncology drug targets, 3 NF-kB (nuclear factor kappa B) complex components (*NFKB1, NFKB2*, and *RELB*) plus *SIGLEC6* and *GSDMD* were included for anti-inflammatory potential, and 2 for metabolic liver disease (*NWD1* and *PNPLA3*). Synucleins A and B and *SORT1* were added as evidence-supported targets in neurodegenerative diseases, and zinc finger transcription factor WIZ was included as a novel Sickle cell disease target. While some new proteins were added as members of existing GtoPdb families, others were added to their own newly created families; e.g. the synuclein proteins (alpha, beta, and gamma) and the NF-kappa B TF proteins (*REL, RELA, RELB, NFKB1*, and *NFKB2*). Whilst we already had a family for the poly ADP-ribosylating PARPs, we created a new family for some of the more well studied mono-ADP-ribosylating PARPs (family members 10, 11, 14, and 15). In relation to coronavirus (CoV) target expansion, to accommodate antiviral ligand coverage, we generated three new protein entries for CoV proteins (nonstructural proteins 10 and 16, and the CoV 2′-*O*-methyltransferase) to allow direct curation of compounds against these viral proteins. *RELA* was added as a human immune system protein that has potential to modify the interferon response to SARS-CoV-2 infection. Nine of the new protein entries have modulators that are in clinical trials: *CLPP, SORT1*, and *STEAP1* have phase three candidates (dordaviprone, latozinemab, and xaluritamig, respectively), *SNCA* has two monoclonal antibodies in phase 2 studies (prasinezumab and cinpanemab), and *ENPP1* has two small-molecule inhibitors in phase 2 (vizenpistat and ISM5939). The remaining four, *NMT1, PNPLA3, LY75*, and *TIPARP* have novel therapeutic agents in phase 1 trials. Four of the 53 new protein targets are unique to the GtoPdb in comparison to target coverage in ChEMBL and BindingDB, and pass the threshold to be included in our submission to UniProt. The four are *ZDHHC3, SORT1, PNPLA3*, and *CDO1*.

### Interaction data

**Figure 2.**
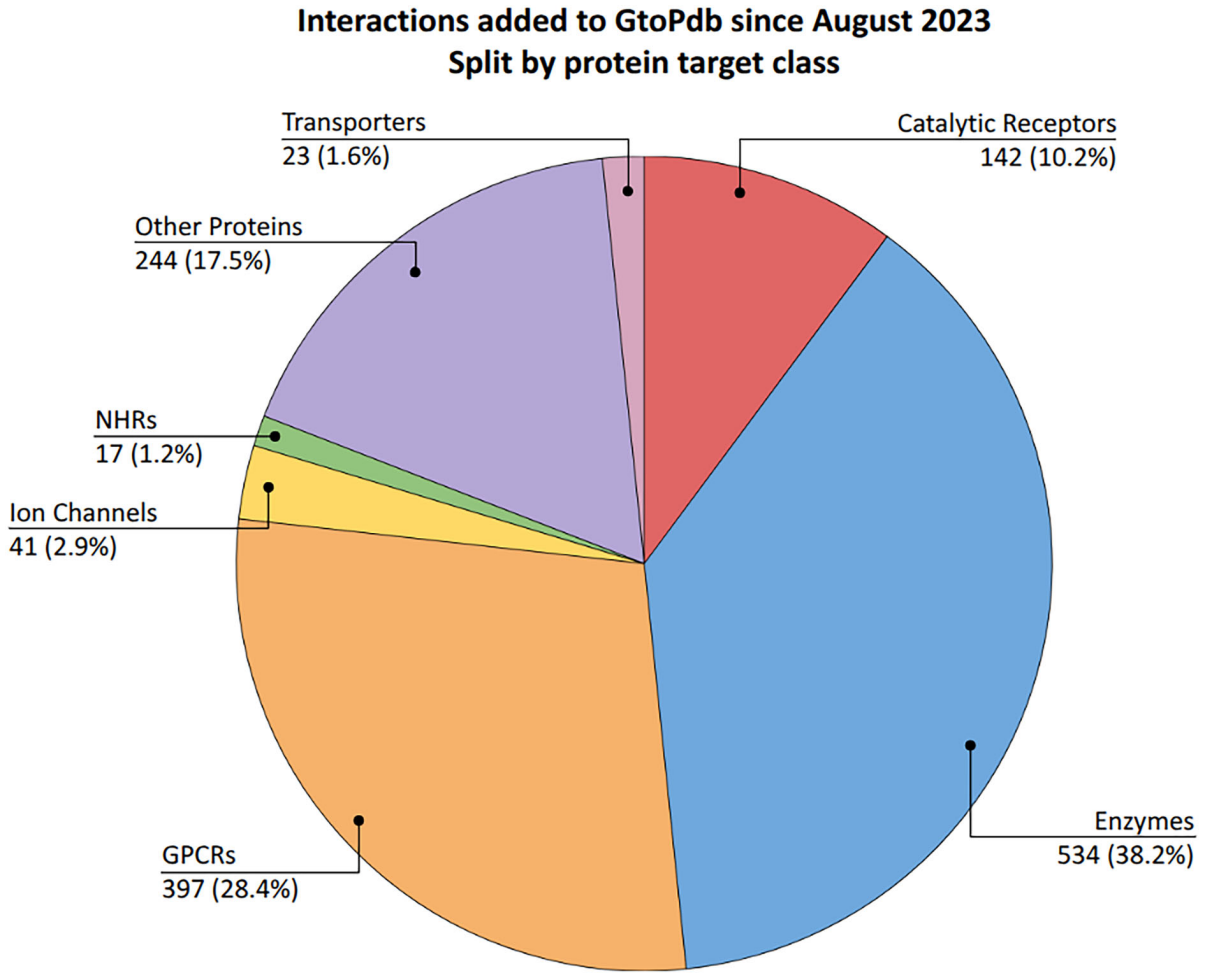
Analysis of new interactions added to GtoPdb since August 2023, split by protein class of the target. Total target–ligand interactions and percentage shown in the labels. Nearly 40% of new interactions have been added to the enzyme class, reflecting the pharmacological interest in the functional role of enzymes in disease. In total 1405 new interactions were added, 1342 of which were for targets with a defined target class (the other were for interactions with ligand targets). In 56 cases, the target is a member of multiple families in different classes so the cumulative count of interactions in this figure is 1398.

**Figure 3.**
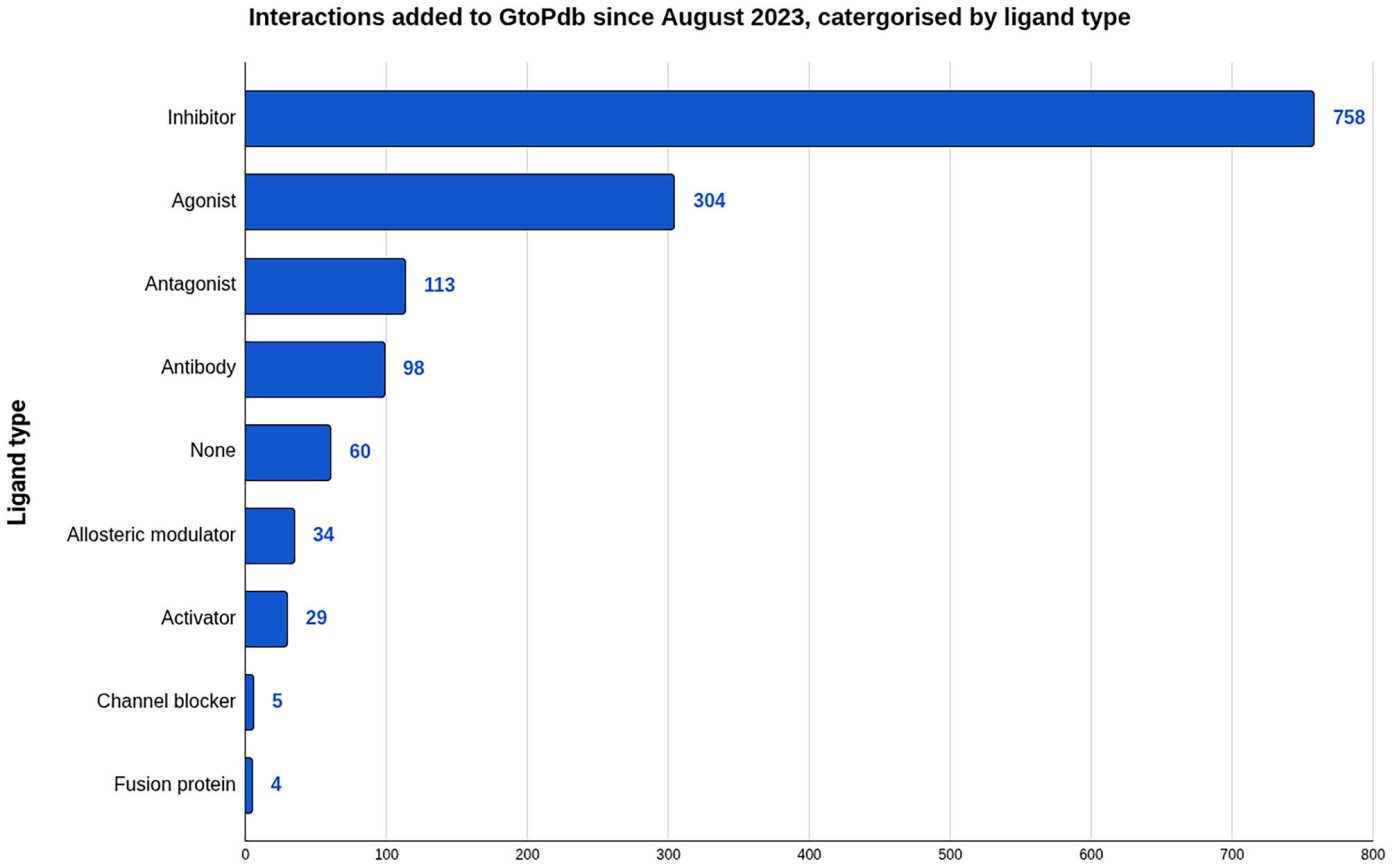
Number of new interactions added to GtoPdb, split by ligand type, since August 2023. Ligand type indicates how the ligand acts in the context of the interaction. “None” indicates no interaction type was assigned by curators. While there is a range of varying ligand types in the newly added interactions, the majority are either inhibitors or agonists, which combined make up ~75% of new interactions. With the number of inhibitors, a reflection on the fact that a majority of new interaction data has been added to enzyme targets.

At the heart of the data in GtoPdb are our curated pharmacological interaction data. Table 1C shows that there is curated ligand interaction for 2027 human protein targets, and 1767 of which have quantitative binding data. Of these, 757 interactions concern approved drugs, 355 of which are where the protein is the primary target of the drug. Curation of pharmacological data concentrates on the most potent and well characterized compounds, typically one from each paper. Since our last update, 1405 new interactions have been added to the database, the majority (1097) for new ligands, but with 308 added for existing ligands. Of the 1405 new interactions, 992 are quantitative. Figure 2 summarizes the new interactions added in the last 2 years, split by protein target class. Five hundred and thirty-four (38.2%) of new interactions have been added for protein targets in the enzyme (534, 38.2%) and GPCR (397, 28.4%) classes. The next highest class is our other proteins class, which has had 244 (17.5%) interactions added. We have quite extensive coverage for protein targets in the enzyme class, particularly for kinases and proteases; therefore, curation can be more weighted toward these families. Additionally, identification of pathological roles for enzymes in disease is creating pharmaceutical interest, and this is reflected in the higher proportion of interactions, in particular inhibitors, added to enzyme protein targets. Figure 3 shows the breakdown of interactions by type, illustrating that a majority of recently added interactions involve inhibitors (758) with the next highest being agonists (304).

### AntibioticDB/GARDP collaboration

ADB (https://www.antibioticdb.com) [13] is a freely available database of antibacterial agents to facilitate research and development of new antibacterial therapeutics. It was originally established in 2017, and since 2021 has been led by the GARDP (https://gardp.org/) [14, 15]. ADBs development was motivated by the need to identify suitable agents for development into new antibacterial drug candidates. In particular that of drug candidates whose original development was discontinued to be revisited and potential developed into successful antibacterial drugs. ADB is continuously updated by mining the published literature and capturing newly discovered antibacterial compounds as they are reported, making ADB a leading global resource on antibacterial agents.

GtoPdb has been collaborating with ADB in projects supported by the GARDP (https://gardp.org/) since 2022. The collaboration has delivered expert-driven oversight to increase the coverage of antibacterial compounds in GtoPdb. In addition, it has brought added value by setting up direct links between GtoPdb ligand summary pages and ADB compound records and provides supplemental chemistry and pharmacology for the antibacterial compounds curated within ADB. During the initial phase of the project, curatorial effort focused on mapping compounds in ADB with existing ligands in GtoPdb, typically done by a combination of name, PubChem CID, and SMILES identifier mapping. This was followed by work to identify antibacterials in ADB that were not already in GtoPdb and to add those that met our threshold for inclusion. Antibacterial ligands continue to be added to GtoPdb, with principal sources that include ADB, peer reviewed literature, and patents. Each new antibacterial ligand entry in GtoPdb is given an “antibacterial” tag and manually curated to include external database links, mechanism of action information where it is known, clinical approval status, and bioactivity data relating to the antibacterial potency of the ligand against pathogens. As indicated in Table 1, there are 668 ligands now tagged in GtoPdb as “antibacterial,” with 183 ligands being added since our last update (Table 2). An initial small set of bacterial protein targets has been curated, with a focus on clinically validated targets and inclusion criteria requiring quantitative pharmacological interaction data. As part of our work in curating antibacterial compounds in GtoPdb, we now have a dedicated landing page for antibacterials (https://www.guidetopharmacology.org/GRAC/AntibacterialsForward). This page consolidates information on antibacterials in GtoPdb, providing information on our collaboration with ADB and giving details about our inclusion criteria for curating antibacterials. The page includes a full table of all the antibacterial ligands curated in the database, with hyperlinks to the specific ligand summary pages for each compound. The table has separate tabs that provide information and links for the curated bacterial protein targets, additional resources and for references and further reading. We continue to work with ADB to support the maintenance and development of their website, with future aims to provide 2D structure display, structure-based searching and improved filter capabilities.

## Guide to PHARMACOLOGY website updates

### FAIR downloads

It remains vital that expert-data resources, such as GtoPdb, provide their data in simple reusable formats thereby ensuring they are FAIR-compliant (Findable, Accessible, Interoperable, Reusable) [33]. The FAIR Principles emphasise improving the ability of machines to automatically find and use data, in addition to supporting its reuse by individuals. We ensure that GtoPdb is FAIR-compliant, so that its data and resources can be of maximum benefit in supporting and improving knowledge for pharmacological research and beyond, and is a named FAIR-sharing resource (https://fairsharing.org/FAIRsharing.f1dv0) [34]. Our download page (https://www.guidetopharmacology.org/download.jsp) provides comma-separated and tab-delimited files (compatible with multiple spreadsheet packages) of GtoPdb targets, ligand, interactions, and more. Across the site, we have also added additional buttons to allow users to download specific datasets. On average, data files are downloaded from GtoPdb ∼690 times a month. We also make available our PostgreSQL file of the entire database. Most recently, we have updated the endogenous/natural ligand pairings file, including UniProtKB and Ensembl IDs for ligands, targets, and subunits where relevant. We have added a more detailed approved drug download, which includes detailed interaction data curated for that ligand. We have also included separate interaction download files for each main target class. The full interaction download is a relatively large file, so splitting by class hopefully makes using the interaction downloads more manageable.

### Nucleic acids

In collaboration with Prof Peter Ferdinandy’s group at the Semmelweis University, Budapest, Hungary, we have modified our curation and display of nucleic acid ligands in GtoPdb. First, there is now a “nucleic acids-based” category available on our ligand list page, https://www.guidetopharmacology.org/GRAC/LigandListForward?type=Nucleic-acid, making this set of ligands easily accessible. We have also revised the display of nucleic acids on the website, with their ligand summary pages now displaying nucleic acid subclasses and targets, along with the nucleic acid sequence and HELM notation (where curated) under the Structure tab. There are 42 nucleic acid-based ligands curated in the database, including all 18 of the currently approved drugs in this class. Unfortunately, for many of these we are unable to provide fully defined, machine-readable chemical structures (SMILES, InChI keys), and are restricted to the available basic nucleic acid sequence information. In line with all of our other approved drugs we curate approval information (dates, indications, trade names etc.). We are continuing to add drugs from this class that are clinical candidates in phase 1–3 development.

### Natural products

NPs are an important source of biologically active compounds and their potential in drug discovery and development is well recognised. However, it is critically important that there is thorough validation of the pharmacology profile of any new NP drug [35]. As such, we have been collaborating with the Italian Society of Pharmacology (SIF) since 2023 in an expert-driven project to improve the curation of NPs as a resource within GtoPdb. The initial phase of this project reviewed all of the ligands that were selected as “NP” in the GtoPdb dataset to determine which were truly NPs. For example, some were either semi-synthetic analogs or derivatives, so these were removed from the NP ligands set. Many of the existing NP ligand pages have been updated either with general comments, or information and references to targets, and interaction data where available. Beyond the initial phase, new ligands that meet the GtoPdb inclusion criteria have been added, supported by regular updates from SIF. Since our last update ∼144 new NPs have been added (Table 2), with 26 being removed from this category as part of the review process. This means there are now a total of 521 NPs curated in GtoPdb. These have been screened from primary medicinal chemistry literature, NP-specific journals, a few from BJP/BJCP author requests and sources such as this 2024 review of GPCR-targeting NPs [36]. The NPs landing page (https://www.guidetopharmacology.org/GRAC/NaturalProductsForward) on the GtoPdb website gives details about our collaboration with SIF and provides definitions of NPs and explains our curation inclusion criteria. The page also shows content statistics for NPs and presents tables of the NPs curated in GtoPdb.

## Guide to PHARMACOLOGY usage and connectivity

### Site usage and impact

GtoPdb is an open-access resource that aims to provide accurate information on the basic science underlying drug action to support research and education. It continues to be well accessed by users, which we track via Google Analytics. In the 12 months to 16 July 2025, the site has seen a monthly average of ∼51 775 sessions (∼43 350 engaged sessions) from ∼32 700 users and an average of 247 079 page views per month. In the same time period, access to the site, although dominated by the USA, China, UK, and India (∼54% of users), has come from a total of 56 different countries (where > 500 sessions were recorded). Our two most recent NAR database updates from 2022 and 2024 [12, 19], according to European PubMed Central impact statistics, have acquired a combined 182 citations and an Altmetric score of 22.

### Links with external resources including PubChem

GtoPdb maintains numerous connections with other resources, many of which are reciprocal in terms of cross-pointing web links. We have a long-standing collaboration with HGNC [37] on shared nomenclature interests and our adherence to mapping HGNC-approved human gene symbols and names to NC-IUPHAR nomenclature. In addition, we have built links between more specialist resources such as GPCRdb [38]. GtoPdb submits data to NCBI via their link-out utility, which indexes our PMIDs (https://pubmed.ncbi.nlm.nih.gov/?term=loprovguidpharm[SB], 33 449 PMIDs) with additional links for genes, nucleotides, and proteins. We also submit to Europe PMC (https://europepmc.org/) through their external links service (https://europepmc.org/LabsLink). Using this service, links are added from Europe PMC articles to related GtoPdb target and ligand data where the article is a reference to a curated pharmacological interaction in GtoPdb. A full list of the 8677 Europe PMC articles with GtoPdb data links (at the time of writing) can be retrieved from https://europepmc.org/search?query=%28LABS_PUBS%3A%221969%22%29. Wherever we have cross-links to other resources, we maintain contacts with the teams concerned and do our best to ensure these are regularly refreshed.

**Figure 4.**
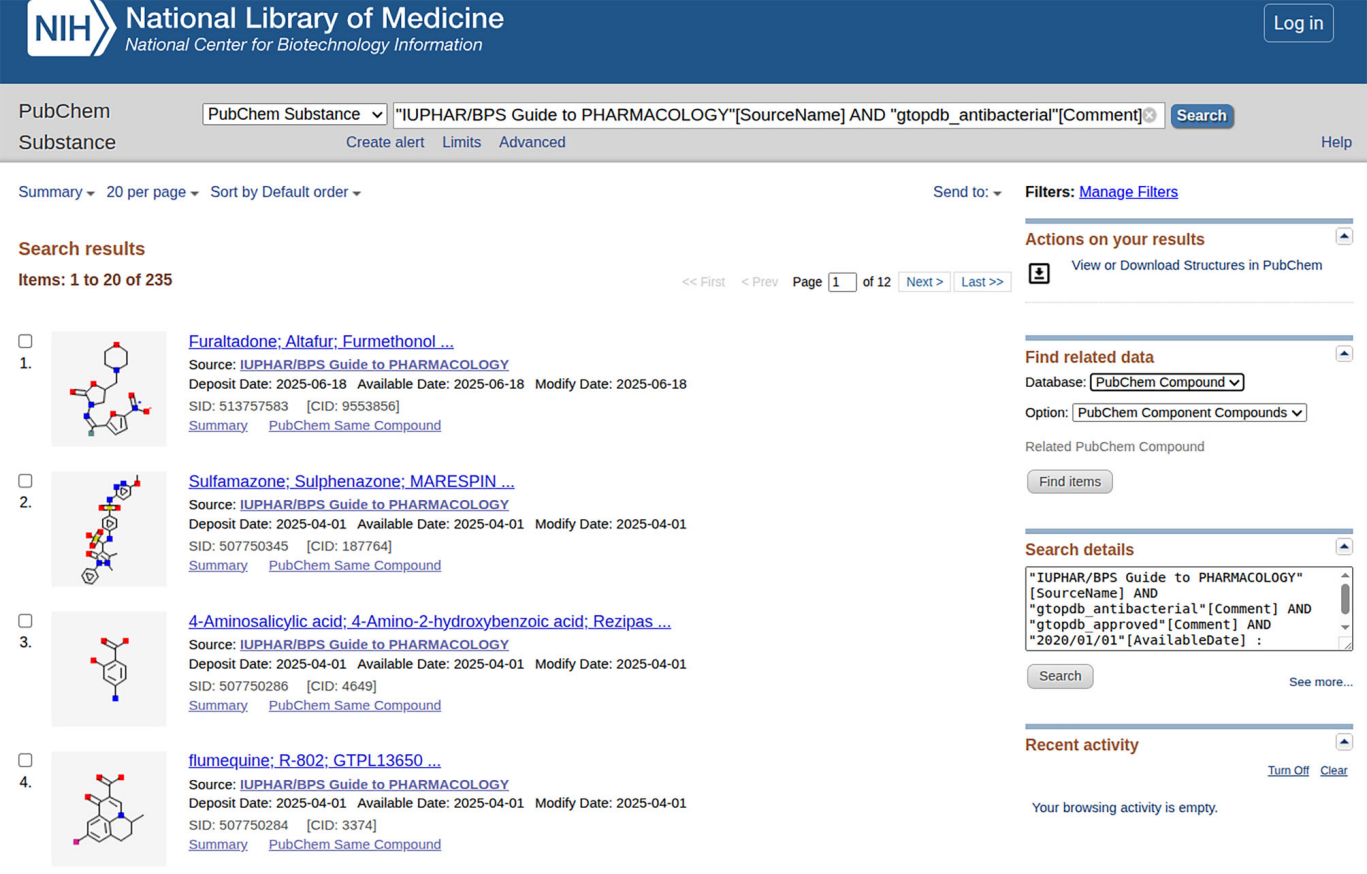
Showing an advanced search result for GtoPdb data in PubChem. The query asks for PubChem substances, for which GtoPdb is a source, made available since 2020, tagged with our approved drug and antibacterial categories. The full query used is (“IUPHAR/BPS Guide to PHARMACOLOGY”[SourceName] AND “gtopdb_antibacterial”[Comment] AND “gtopdb_approved”[Comment] AND “2020/01/01″[AvailableDate] : “3000”[AvailableDate]).

Notable are our links with PubChem [20] to which we submit substances after each database release. The value of GtoPdb ligand submission and integration into PubChem [20] and other NCBI resources [39], have already been described [6, 19]. When our ligands are submitted to PubChem, they are each assigned a unique PubChem Substance ID (SID). SIDs are community-submitted structures with information about the molecule. PubChem merges unique chemical structures from different SIDs as PubChem Compounds (CIDs). As mentioned earlier, one of the strengths of GtoPdb is its regular release cycle, with new database releases approximately every quarter. As a consequence, GtoPdb can often be the first submitter of a substance to PubChem. For example, a query of PubChem for compounds where IUPHAR/BPS GtoPdb is the source and where there is only one depositor, show 42 compounds as of 18 August 2025 (https://www.ncbi.nlm.nih.gov/pccompound/?term=%22IUPHAR%2FBPS±Guide±to±PHARMACOLOGY%22%5BSourceName%5D±AND±0%3A1%5BDepositorCount%5D). Around half of these are compounds added to PubChem following our 2025.2 release (18 June 2025), as indicated by the “Created Date.” As part of the submission process, we add depositor substance comments which can then be exploited via the PubChem search interface. We “tag” our depositor comments with various GtoPdb ligand categories, which are then able to be used as filters within the PubChem query interface (https://www.ncbi.nlm.nih.gov/pccompound). Table 3 shows the selections of five different tags, indicating how many PubChem SID and CIDs are tagged in this way. By way of illustration, the screenshot in Fig. 4 shows the result of the combined query “approved drug,” “antibacterial,” and “available after 1 January 2020.” A more detailed illustration of how these tags can be exploited is described in more detail by Dr. Chris Southan in this blog post (https://cdsouthan.blogspot.com/2022/11/guide-to-pharmacology-selectable.html) and in this more recent post (https://cdsouthan.blogspot.com/2024/11/exploiting-minable-connectivity-from.html).

**Table 3.** Counts of GtoPdb tagged ligands in PubChem (July 2025), using selected substance queries (column 2)

Ligand type	Query^a^	SID count	CID count
Approved drugs	gtopdb_approved [comment]	2120	1833
Immunopharmacology	gtopdb_immuno [comment]	1531	1057
Antimalarial	gtopdb_malaria [comment]	143	140
Antibacterial	gtopdb_antibacterial [comment]	668	654
Antibody	gtopdb_antibody [comment]	354	0
Natural products	gtopdb_natural_product [comment]	521	514

^a^Example query format for approved drugs ‘“IUPHAR/BPS Guide to PHARMACOLOGY”[SourceName] AND “gtopdb_approved”[comment]’

CID numbers are retrieved via the “Find related data” > “Database” > “PubChem Compound” > “PubChem Same Compound” > CID count and display (i.e. a SID > CID conversion).

### Comparing GtoPdb with other resource

**Figure 5.**
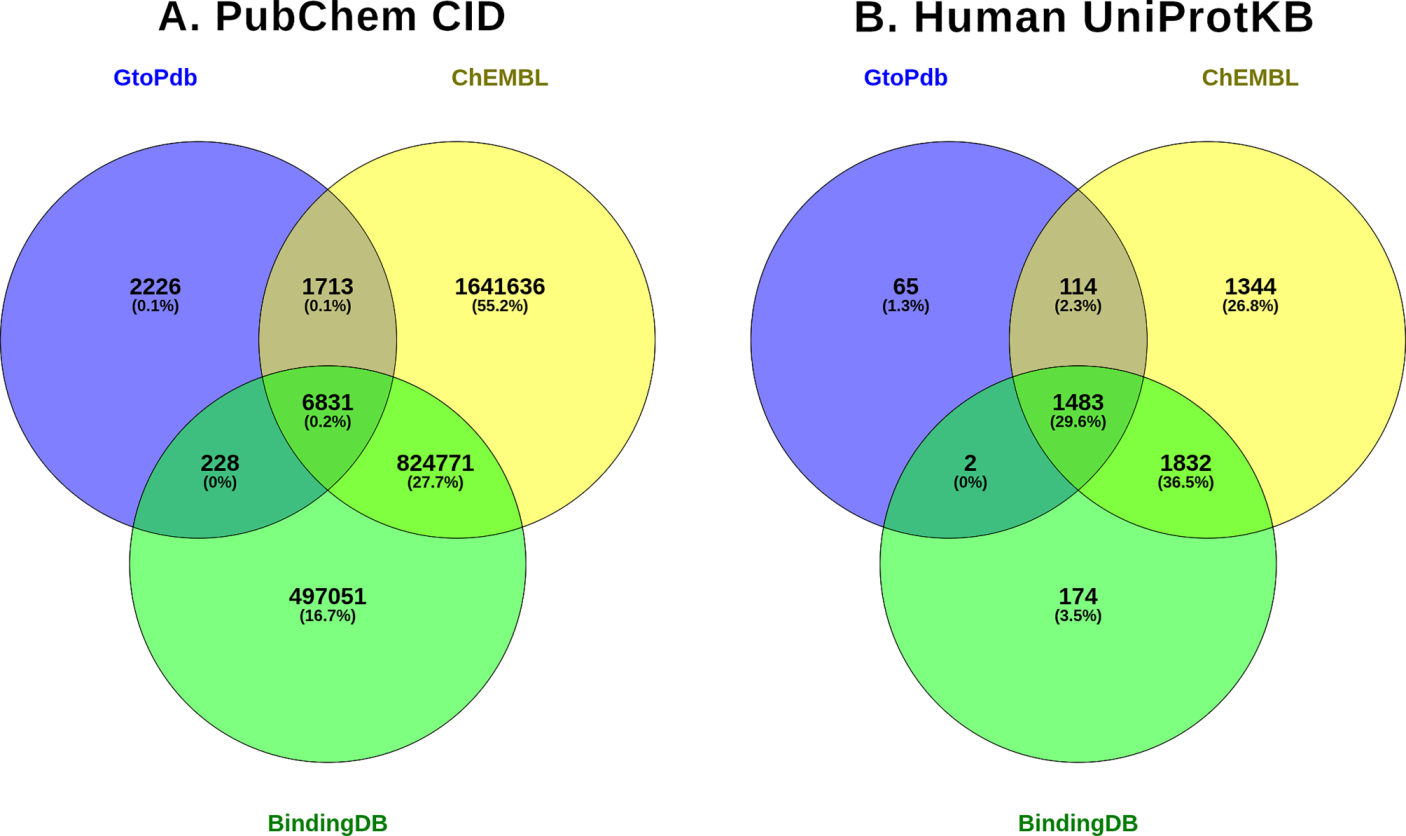
Venn diagrams showing the intersections of PubChem CIDs (**A**) and Human UniProtKB IDs (**B**) between GtoPdb, ChEMBL and BindingDB. CID counts are taken using the advanced PubChem Compound search (https://www.ncbi.nlm.nih.gov/pccompound), specifying source name in the query (i.e. “IUPHAR/BPS Guide to PHARMACOLOGY”[SourceName]. UniProtKB counts are taken from the UniProtKB advanced search, filtering on Cross-Reference > Chemistry Database (i.e. https://www.uniprot.org/uniprotkb?query=%28database%3Aguidetopharmacology%29±AND±%28organism_id%3A9606%29±NOT±%28database%3Achembl%29). Note, the number of 1664 GtoPdb Human UniProtKBs (1664) is lower than the total in GtoPdb because we only make available targets with quantitative affinity values above 6 for UniProt to include in the cross-referenced database set. Diagrams prepared using Venny (https://bioinfogp.cnb.csic.es/tools/venny/).

To illustrate where GtoPb sits in the ecosystem of pharmacological resources we show here the intersections, differences and complementarity with ChEMBL [25] and BindingDB [32]. While there is variation in data models of the individual resources and how they can be queried, content comparison is possible as all three submit to PubChem and have target cross-references in UniProtKB. Figure 5 shows a Venn diagram comparing PubChem CIDs and UniProtKB between GtoPdb, ChEMBL and BindingDB. Around 22% of GtoPdb compounds do not overlap with ChEMBL or BindingDB. ChEMBL extracts all assay data, including ADMET determinations, from a paper whereas GtoPdb usually extracts just the lead compound but will also curate reported secondary target activity. In the comparison with BindingDB, 36% of GtoPdb compounds do not overlap. BindingDB’s uniqueness is mainly their patent curation; it also has an arrangement with ChEMBL from which it subsumes just the individual protein target-mapped data. GtoPdb human target overlap with both ChEMBL and BindingDB is extensive; GtoPdb has 67 human protein targets not in ChEMBL and 179 not in BindingDB.

### Future directions

Maintain our high-standard of curation by tracking the inclusion of pre-prints in the database and whether a subsequent reviewed paper is published or retracted, as discussed earlier (retractions paragraph). The GtoPdb remains an open-access and freely available resource, but unfortunately, the current funding landscape threatens our ability to ensure GtoPdb’s sustainability going forward. We do ask that any commercial organisations that use GtoPdb to take the opportunity to support us (https://www.guidetopharmacology.org/gtopdbSustainability.jsp) and/or sponsor the GtoPdb, if interested please get in touch at equiries@guidetopharmaoclogy.org.

### Data access

GtoPdb, GtoImmuPdb, and GtoMPdb are available online at https://www.guidetopharmacology.org, https://www.guidetoimmunopharmacology.org and https://www.guidetomalariapharmacology.org, respectively. All three resources are licensed under the Open Data Commons Open Database License (ODbL) (https://www.opendatacommons.org/licenses/odbl/), and the contents are licensed under the Creative Commons Attribution ShareAlike 4.0 International (CC BY-SA 4.0, https://creativecommons.org/licenses/by-sa/4.0/). Advice on linking to us and for accessing and downloading data are provided here: https://www.guidetopharmacology.org/linking.jsp. GtoPdb makes three to four public database releases per year; the data summaries and statistics reported in this paper are from release 2025.2 (June 2025). Our downloads page (available from https://www.guidetopharmacology.org/download.jsp) provides a dump file of the full PostgreSQL database, in addition to several specific download files for targets, ligands, interactions, peptides, endogenous/natural ligands, and approved drugs with primary targets, ligand ID mapping, ligand SDF file, and RDF flat files. Our REST web services are available at https://www.guidetopharmacology.org/webServices.jsp and provide computational access to data in JavaScript Object Notation (JSON) format.

### Citing the resource

This publication replaces all previous papers for citing this resource. Citation advice for specific target pages appears on the website. Please refer to our resources on first mention by full correct name (IUPHAR/BPS Guide to PHARMACOLOGY, IUPHAR Guide to IMMUNOPHARMACOLOGY, IUPHAR/MMV Guide to MALARIA PHARMACOLOGY), including the capitalization. For subsequent abbreviations, please use GtoPdb, GtoImmuPdb, and GtoMPdb, specifying the release version number, which can be found on our About page (https://www.guidetopharmacology.org/about.jsp#content).
